# Experimental Cowpox Virus (CPXV) Infections of Bank Voles: Exceptional Clinical Resistance and Variable Reservoir Competence

**DOI:** 10.3390/v9120391

**Published:** 2017-12-19

**Authors:** Annika Franke, Rainer G. Ulrich, Saskia Weber, Nikolaus Osterrieder, Markus Keller, Donata Hoffmann, Martin Beer

**Affiliations:** 1 Institute of Diagnostic Virology, Friedrich-Loeffler-Institut, Federal Research Institute for Animal Health, 17493 Greifswald-Insel Riems, Germany; franke.annika@gmail.com (A.F.); Saskia.Weber@fli.de (S.W.); Martin.Beer@fli.de (M.B.); 2 Institute of Novel and Emerging Infectious Diseases, Friedrich-Loeffler-Institut, Federal Research Institute for Animal Health, 17493 Greifswald-Insel Riems, Germany; Rainer.Ulrich@fli.de (R.G.U.); Markus.Keller@fli.de (M.K.); 3 Institute of Virology, Freie Universität Berlin, 14163 Berlin, Germany; no.34@fu-berlin.de

**Keywords:** Cowpox virus, reservoir, host, voles

## Abstract

Cowpox virus (CPXV) is a zoonotic virus and endemic in wild rodent populations in Eurasia. Serological surveys in Europe have reported high prevalence in different vole and mouse species. Here, we report on experimental CPXV infections of bank voles (*Myodes glareolus*) from different evolutionary lineages with a spectrum of CPXV strains. All bank voles, independently of lineage, sex and age, were resistant to clinical signs following CPXV inoculation, and no virus shedding was detected in nasal or buccal swabs. In-contact control animals became only rarely infected. However, depending on the CPXV strain used, inoculated animals seroconverted and viral DNA could be detected preferentially in the upper respiratory tract. The highest antibody titers and virus DNA loads in the lungs were detected after inoculation with two strains from Britain and Finland. We conclude from our experiments that the role of bank voles as an efficient and exclusive CPXV reservoir seems questionable, and that CPXV may be maintained in most regions by other hosts, including other vole species. Further investigations are needed to identify factors that allow and modulate CPXV maintenance in bank voles and other potential reservoirs, which may also influence spill-over infections to accidental hosts.

## 1. Introduction

Over the past 15 years, many new viruses and known viruses have (re-)emerged and are frequently causing zoonotic diseases [1,2]. The viruses are either transmitted to humans from non-human vertebrates (vertebrate-borne diseases) or by arthropods (vector-borne diseases). Cowpox virus (CPXV) is a zoonotic pathogen known to circulate among rodents in Europe [1]. Human CPXV infections are relatively rare [3,4,5,6,7,8,9] and CPXV usually causes a self-limiting disease in humans, predominantly lesions on hands or face [3,4,5,6,7,8,9]. However, in immunocompromised patients, CPXV infections can readily generalize and result in severe and sometimes lethal infections [4,5].

The species *Cowpox virus* belongs to the genus *Orthopoxvirus* (OPV), subfamily *Chordopoxvirinae*, family *Poxviridae*. CPXV is endemic in Europe and Northern and Central Asia [10,11]. Many mammal species are known to be susceptible to CPXV infection, among them cats [6,12], rats [7,8,9], alpacas [13], elephants [14], and primates such as cotton-top tamarins [15]. Cats seem to be the main source of human CPXV infections, although wild rodents, primarily voles, are believed to be the definitive reservoir hosts for the virus [10,11].

Investigations on wild rodents as potential reservoir hosts of CPXV started in England in the 1980s. Until now, serological surveys indicating CPXV infections in wild rodents have been reported for several countries of Eurasia: The United Kingdom [16,17,18], Belgium [19], Finland [20,21], Norway [22], Germany [21,23], Turkmenia [24], Vietnam [25], Georgia [26] and Hungary [27]. Here, voles (bank vole, *Myodes glareolus* [18,19,21,27], field vole, *Microtus agrestis* [17,21]), and murine rodents such as the striped field mouse, *Apodemus agrarius* [21], wood mouse, *Apodemus sylvaticus* [19,22], and Norway rat (*Rattus norvegicus* [22]), tested positive for CPXV-specific antibodies. Bank voles were shown to reach maximum seroprevalence of 71% in Hungary [27], 64% in Belgium [19], and 72% in the UK [18].

In the UK, different field studies indicated the circulation of CPXV in rodents and demonstrated peaks of infections in bank voles and wood mice, although interspecies transmission was negligible [28]. Correlations of CPXV infection and vole survival [29] or interactions of CPXV and other microparasites in simultaneously infected voles were observed [30]. First experimental infections in the late 1990s revealed that young bank voles (three to five weeks old) developed antibodies between 10 and 14 days post infection (dpi) independently of the inoculation route (CPXV strain L97; intradermal, subcutaneous or oronasal) [31]. In addition, Feore et al. reported that CPXV infections of bank voles reduced fecundity by increasing the time to first litter [32].

However, CPXV has not yet been isolated from vole or mice species other than the common vole (*Microtus arvalis*) [33]. As isolation is one of the criteria supporting the identification of a species being a natural reservoir of a certain pathogen (according to [34]), the role of bank voles in central Europe for CPXV epidemiology is doubtful. Post-glacial colonization of Europe by bank voles from different refuges resulted in the establishment of different evolutionary lineages, with the Western, Eastern and Carpathian lineages in Central Europe [35,36]. The experimental inoculation of the supposed reservoir species resulting in infection and shedding is a criterion that needs to be met for a natural reservoir definition [34]. Our recent infection experiments showed susceptibility of common voles to oronasal CPXV infection, which also resulted in respiratory symptoms and virus excretion [33]. We, therefore, decided to perform similar experimental inoculations with bank voles of different evolutionary lineages and age groups to further determine their potential as putative CPXV reservoir species. The CPXV isolates used here originated from different geographical origin, from accidental hosts (human, rat or cat), and also from one reservoir host species, the common vole. With the polyphyletic nature of the species *Cowpox virus* in mind, members of four CPXV clades (according to [37]) were used. In addition, CPXV was applied by different inoculation routes.

## 2. Materials and Methods

### 2.1. Viruses

CPXV strains of different origins (summarized in Table 1) were propagated on Vero76 cells (Collection of Cell Lines in Veterinary Medicine (CCLV), Friedrich-Loeffler-Institut, Greifswald-Insel Riems, Germany).

### 2.2. Animals

Outbred bank voles (*Myodes glareolus*) originated from in house-breeding and were kept under standardized conditions: type III cages; 22 °C; 12/12 h light cycle, ≈60% humidity; water and rodent pellets ad libitum as the diet. The specific pathogen-free status with regard to CPXV of the breeding colonies was controlled on a regular basis by serological assays. The breeding colonies originated from voles of the Western evolutionary lineage, provided by the Federal Environmental Agency in Berlin, Germany, and voles of the Carpathian evolutionary lineage, provided by Jagiellonian University Krakow, Poland. PCR amplification and sequencing of the partial cytochrome *b* gene following a standard protocol [42] confirmed the different evolutionary lineages (data not shown).

### 2.3. Infection Experiments and Sampling

The animal experiments were evaluated by the responsible ethics committee of the State Office for Agriculture, Food Safety and Fishery in Mecklenburg-Western Pomerania (LALFF M-V) and governmental approval was obtained (registration number 7221.3-1.1-020/13, 27 May 2013). The design of all experiments is summarized in Table 2. Initially, we inoculated bank voles of the Western lineage with seven CPXV strains originating from different host species (Table 1 and Table 2, experiment #1). The voles were of mixed ages (3 to 4 months or 1-year-old) and mixed sex. Virus was given intranasally at 10^5^ TCID_50_/animal. Body temperature, weight, and general health status were checked daily over a period of 21 days. In addition, nasal swabs were taken every other day until 21 dpi by applying a wetted swab onto the rhinarium of the individual vole. Some animals were euthanized for autopsy on 5 dpi or 21 dpi, when different organ samples (rhinarium and nasal epithelia, skin, liver, lung, spleen, trachea) and blood were collected individually.

Further experiments were done to examine the influence of the application route, the age and origin of the voles (Table 2, experiments #2–#5). As indicated, in-contact animals were grouped together with CPXV-inoculated animals in some experiments. After 24 h of separation, contact voles were caged together with CPXV-inoculated animals to determine transmission potential. All nasal swabs were directly suspended in 2 mL cell culture medium (mixture of equal volumes of Eagle MEM (Hanks’ balanced salts solution) and Eagle MEM (Earle’s balanced salts solution), 2 mM l-Glutamine, nonessential amino acids, adjusted to 850 mg/L NaHCO_3_, 120 mg/L sodium pyruvate, 10% fetal bovine serum (FBS), pH 7.2; suited for closed tissue culture vessels and incubation under 2.5% CO_2_ atmosphere and supplemented with antibiotics: 1% enroflaxin (Bayer, Leverkusen, Germany), 0.5% lincomycin (WDT, Garbsen, Germany) and 0.2% amphotericin/gentamicin (Gibco Life technologies, Carlsbad, Germany). The organ samples were placed into reaction tubes of 1 mL cell culture medium (see above) supplemented with 1% penicillin-streptomycin and a stainless-steel bead (5 mm diameter).

### 2.4. Analysis of the Samples

Viral DNA loads of all samples were determined by quantitative PCR (qPCR) using OPV-specific primers [43]. Organ tissues were homogenized (TissueLyser II; Qiagen; Hilden, Germany). DNA extraction was done semi-automatically by the BioSprint 96 instrument (Qiagen) using the NucleoMag VET kit (Macherey-Nagel, Düren, Germany). The sera were analyzed by an indirect immunofluorescence (IF) assay to detect OPV-specific antibodies. In brief, serum samples were first inactivated for 30 min at 56 °C. Subsequently, CPXV-infected Hep2 cells (fixed with methanol-acetone at 1:1 and incubated with Tris-buffered saline plus Tween (TBS-T) for 30 min) were incubated for 1 h at room temperature with different serum dilutions (1:20, 1:40, 1:80, 1:160, 1:320). After three washing steps with PBS, a commercial anti-mouse secondary antibody conjugated to Alexa488 (Thermo Fisher Scientific, Waltham, MA, USA) was applied. The cells were visualized under a fluorescence microscope. The titer was taken as reciprocal of the greatest serum dilution, which showed positive detection and those animals with reaction at titers of ≥1:40 were considered positive.

### 2.5. Statistics

Results were statistically evaluated per groups by ANOVA using SPSS (IBM, Ehningen, Germany). The Tukey HSD test (*p* = 0.05) was performed (SPSS) to determine whether results were significantly different between groups.

## 3. Results

### 3.1. CPXV Infection of Bank Voles of the Western Evolutionary Lineage with Different CPXV Strains Induced no Clinical Signs (Experiment #1)

The initial infection experiment (Table 2, experiment #1) did not result in clinical signs when any of the Western lineage bank voles were inoculated intranasally with different CPXV strains. In addition, body weight and body temperature were stable for all animals for the duration of the observation period (data not shown). Most animals developed antibodies, but with varying titers (Table 3, Table S1). Inoculation with the reference CPXV strain Brighton Red or the CPXV isolate FIN_MAN_2000 induced anti-CPXV antibodies in all animals and resulted in the highest antibody titers (up to 1:320, Table S1). In contrast, in the group inoculated with the common vole-derived CPXV strain Ger/2007/vole, only one individual developed antibodies with a low titer of 1:20 (Table S1). Statistical evaluation of antibody titers revealed significant differences of the seropositivity in animals inoculated with Brighton Red compared to Ger 91/3 and Ger/2007/Vole (Table 3). In addition, antibody titers in animals inoculated with FIN_MAN_2000 differed significantly from those in voles inoculated with RatPox09, Ger 91/3 and Ger/2007/Vole (Table 3). The other group comparisons showed no significant differences (*p* > 0.05).

The distribution of virus DNA in different organs was tested by qPCR and the results are summarized in Table 4. On five dpi, viral DNA was detected in the rhinarium and in the trachea in nearly all animal groups (except the voles inoculated with CPXV RatPox09). In addition, in two animals inoculated with CPXV Brighton Red or FIN_MAN_2000, respectively, the lungs also scored positive for viral DNA. Besides the respiratory tract, CPXV DNA could also be found in the skin (1 x CPXV Brighton Red, 1 x CPXV Ger 91/3). Organ samples from autopsy at 21 dpi were all negative (data not shown). Furthermore, no viral shedding was detected in the CPXV-inoculated animals over a period of 21 days (data not shown).

### 3.2. Intranasal Inoculation of Western Lineage Bank Voles with RatPox09 Induced a Stronger Antibody Response than Subcutaneous Inoculation (Experiment #2)

The second experiment (Table 2) was limited to the CPXV strain RatPox09 in order to compare the outcome with our previous studies using the same strain in both rats [39] and common voles [33].

Two different application routes (intranasal, as used in experiment #1, and subcutaneous) were tested. Bank voles were checked daily and nasal swabs were taken over a 14-day period. None of the inoculated animals showed any clinical signs and all swab samples as well as all organ samples scored negative by qPCR. Serum antibodies were detected with the IF test (Table 5), with titers up to 1:320 (Table S2). Generally, voles inoculated intranasally developed significantly higher antibody titers than voles inoculated subcutaneously (*p* = 0.05; Table 5 and Table S2). One contact animal, which was housed together with the intranasally inoculated animals, also produced high titers of OPV-specific antibodies (Table S2).

### 3.3. The Evolutionary Bank Vole Lineage Had no Influence on the Results of Intranasal Inoculation with RatPox09 (Experiment #3)

In order to evaluate potential influences of the evolutionary lineage, young voles at an age of four weeks from the Western and Carpathian lineage were inoculated intranasally with CPXV RatPox09 (Table 2, experiment #3). None of the animals excreted virus over a period of 14 dpi as proven by negative swab samples (data not shown). There was also no viral DNA detected in the organ samples (data not shown), and the voles produced only low amounts of antibodies with a titer of up to 1:80 (Table S3) that did not differ significantly between the two vole lineages. In addition, none of the contact animals showed any signs of infection or seroconversion. Interestingly, with the restriction that the results were generated in two independent experiments (experiment #2 and experiment #3), younger bank voles exhibited significant (ANOVA analysis, *p* value of 0.00029) lower seroconversion rates compared to adult bank voles (greater than four weeks of age).

### 3.4. Footpad Inoculation Induced No Clinical Signs, but a Strong Antibody Response, Independent of CPXV Strain and Bank Vole Evolutionary Lineage (Experiment #4)

Next, we investigated the footpad inoculation route, which is widely used in *Vaccinia virus* (VACV) trials. Bank voles from both lineages (all younger than three months) were inoculated via footpad with either CPXV RatPox09 or CPXV FM2292 (Table 2, experiment #4). However, neither viral shedding in the nasal swabs nor viral DNA in the organ samples could be detected (data not shown); however, CPXV-specific antibodies were detected in almost all inoculated animals with titers reaching 1:320 for single animals in each group (Table S4), irrespective of the lineage origin of the individual (data not shown). In addition, one contact animal, housed together with CPXV FM2292-inoculated voles, seroconverted with a high antibody titer (Table S4). Nevertheless, seroreactivity did not differ significantly (ANOVA analysis) between animals inoculated with either virus strain after footpad inoculation (Table 6).

### 3.5. Repeated Inoculations with Different CPXV Strains Resulted in Subclinical Infection with a Strong Antibody Response (Experiment #5)

Finally, a possible booster effect of repeated inoculations was investigated. Therefore, bank voles were inoculated intranasally with either CPXV Brighton Red, CPXV FM2292 or CPXV RatPox09. These strains were selected for this experiment as Brighton Red and FM2292 belong to the same clade CPXV-like 2 (Table 1), while FM2292 and RatPox09 were both isolated from a rodent origin sample. At 21 dpi the initial inoculation was followed by a second intranasal application of the same virus strain (Table 2, experiment #5). Swabs were analyzed over a period of 42 days, but no viral DNA could be detected (data not shown). Half of the CPXV RatPox09-inoculated voles seroconverted. In contrast, all animals inoculated with CPXV Brighton Red or CPXV FM2292 developed OPV-specific antibodies reaching higher levels (Table S5). Statistical evaluation revealed significant differences only between the groups inoculated with CPXV Brighton Red and CPXV RatPox09 (Table 7).

## 4. Discussion

It has been reported that wild rodents are the reservoir hosts for CPXV [10,11,28], and we recently demonstrated the susceptibility of common voles for CPXV by experimental infection [33]. However, bank voles evidently are also affected as shown by serological and molecular surveys in Eurasia [16,17,18,19,20,21,22,23,25,26,27]. In addition, recent PCR investigations indicated CPXV infections in bank voles mainly of the Western lineage, but also in a single animal of the Eastern lineage ([21]; Fischer, Drewes, Ulrich et al., unpublished data). Nevertheless, in these cases, the genome load was very low and CPXV could not be isolated. Still, little is known about the pathogenesis of CPXV infections in potential reservoir hosts including the bank vole. We therefore conducted a series of experimental infections of bank voles to investigate the susceptibility to CPXV infection in this potential reservoir host, and compared the data to those from recent experiments with common voles [33].

Bank voles from two different evolutionary origins (Western and Carpathian lineage) were infected with various CPXV strains originating from either accidental or natural host species and belonging to different genetic clades (Table 1). Different inoculation routes were used ranging from intranasal to subcutaneous and footpad applications. The experimental layout was limited by the animal numbers available at any one time and, therefore, resulted in several independent experiments. In addition, the available animals were outbred, which may also account for variability between the individual experiments. However, we contend that general patterns of infections are deducible from our experiments and a clear picture emerged concerning the clinical outcome.

### 4.1. Bank Voles Are Resistant to CPXV-Induced Clinical Signs

Of note, not a single bank vole in the experiments conducted here exhibited any clinical symptoms, although wild rodents have been reported to exhibit clinical signs [24,33,44]; therefore, the asymptomatic course of infection observed here is exceptional. The outcome was independent of the CPXV strain used, the age, the sex, the inoculation route and the bank vole lineage. Our observations support the results of experimental infection of British vole species performed by Bennett et al., which also resulted in subclinical infection [31]. In conclusion, bank voles seem to be one of the most resistant species for CPVX-induced clinical signs.

### 4.2. CPXV Replication and Shedding Is Very Limited in Bank Voles

Viral shedding was not detected by nasal and buccal swab testing (irrespective of age, sex, virus strain, inoculation route and host lineage), and transmission as evidenced by seroconversion occurred in only 2 out of 18 contact animals (sum of contact animals from all five experiments performed here). These findings contradict previous studies with a different vole species in which we showed that experimentally infected common voles (*Microtus arvalis*) were clinically affected and excreted virus between 4 dpi and 14 dpi via respiratory secretions [33]. Sensitivity of the diagnostic tests were demonstrated in the previous study and therefore did not contribute to low score genome detection. The possible transmission route between individual bank voles remains elusive, and respiratory transmission seems unlikely. Shedding via urine and feces was reported from experimentally infected rats [45], and might be a limited source of infectious virus also for naïve bank voles in the used experimental setup. It cannot be excluded either that shedding below the detection limit might be sufficient to infect in-contact animals, albeit irregularly. As CPXV has high tenacity [46], contaminated materials for example grass or hay, may function as fomites and might be the epidemiological connection resulting in maintenance of the pathogen in their environment.

It is worth noting that not all inoculated animals seroconverted and only some bank voles had titers above 1:80. Even sequential inoculation did not result in seroconversion of all individuals, which indicates that the adaptive immune system of bank voles may not be necessary to control CPXV infection. This is especially true for individuals inoculated with virus strains originating from central Europe (e.g., CPXV RatPox09 or CPXV/2007/Vole). However, there were some differences detected in bank voles infected with isolates from either Great Britain or Finland (CPXV Brighton Red or CPXV FIN_MAN_2000) compared to the other viral strains used: positive genome loads in the lungs (at least for Brighton Red considered as replicating virus) and 100% seroconversion rates that also were concomitant with higher titers. Strikingly, these virus isolates originate from locations were bank voles, but not common voles, are present. The CPXV-specific seroconversion, therefore, suggests that bank voles constitute as a possible reservoir host. Generally, common voles have a large geographic range extending from Spain across much of Western, Central and Eastern Europe all the way to the Middle East and central Russia [47]. In contrast, common voles are not found in most parts of southern Europe, Fennoscandia, Northern Russia, Iceland and the British isles (apart from the Orkney-islands) [47].

One might speculate from our observation (strains from UK and Finland induce 100% seroconversion in bank voles, that are endemic in UK and Finland) that CPXV strains might be better adapted to a certain main reservoir host in a given geographic region. As a possible consequence, virus strains of Central European origin are mainly adapted to the common vole as reservoir host, while virus strains in Britain or Fennoscandia are more adapted to bank voles. Consequently, in order to obtain virus isolates originating from reservoir host species, common voles should be sampled in Central Europe, while bank voles/field voles and wood mice are species to be sampled in UK and Fennoscandia, respectively. Seroprevalence and molecular survey data indicate CPXV infections are also occurring in bank voles in Central Europe (Belgium [19], Hungary [27]; Kinnunen 2011; our unpuplished data). However, these studies categorized sera as “positive” if titers were 1:20 or higher and could be the result of inefficient replication without efficient transmission. The low genome copy numbers in the very few PCR-positive bank voles in those field studies also support this and are in line with the here reported course of experimental infection. From the comparative data presented here, a robust “cut-off” value of at least 1:40 for scoring sera as reactive against OPV is suggested and may change the number of seropositive animals in field studies.

### 4.3. Bank Voles as CPXV Reservoir Host

From the results of our extensive infection experiments we conclude that, on the basis of the basic minimal definition of a reservoir host as “being a host that transmits, but is not diseased”, bank voles could indeed present a reservoir host of CPXV, although with inefficient transmission to other voles. However, the more precise definition of Haydon et al. [48], defining a reservoir species as “one or more epidemiologically connected populations or environments in which the pathogen can be permanently maintained and from which infection is transmitted to the defined target population”, is more challenging. Following this definition, the role of bank voles as a general reservoir host for CPXV is questionable since the maintenance of the pathogen in a population is a prerequisite for a reservoir host species. Our studies indicated only limited transmission to contact animals, ergo facilitating limited maintenance. In contrast to a one host species–one virus association as specified, e.g., for hantaviruses [49], CPXV strains might be therefore maintained by multiple species reservoirs. In our opinion, there is generally no unique reservoir host of CPXV rather than a favorite vole species taking the part of the reservoir within a given geographic region.

The competence of a certain vole species to act as reservoir is dependent on host factors as was shown for the cycling of *Borrelia* ssp. in voles and ticks [50]. Additional factors, including co-infections with bacteria or parasites and a general immunosuppression, might be additive, which will have to be tested in future experimental setups. Turner et al. analyzed interactions between microparasite species in field voles and demonstrated that 79% of CPXV-infected animals were co-infected with either *Bartonella* spp., *Anaplasma* spp. or *Babesia* spp. [29]. Furthermore, stress and fecundity are also most likely important key factors playing a role in the kinetics of viral replication in voles. Regarding seroconversions and some hematological parameters, studies of Beldomenico et al. showed in field voles that a poor body condition significantly increased the probability of CPXV-infection, especially for males [30].

In general, experimental infections of reservoir host species are a prerequisite for the dynamic modeling of infectious (zoonotic) diseases. Virus tropism obviously differs between natural reservoirs and accidental host species, and we posit that reservoir studies as conducted here are fundamental. Future studies will have to focus onto the identification of viral genetic markers involved in the interaction of the reservoir host species and their “matching” CPXV strains. In addition, bank vole host factors influencing the level of CPXV replication will have to be analyzed in more detail, and we propose to particularly evaluate the contribution of co-infection and immunosuppression. Finally, the factors preventing bank voles from clinical signs after CPXV infection should be studied, including the role of innate immunity.

## Figures and Tables

**Table 1:** Characterization of CPXV strains used for experimental infections.

Isolate (Accession Number)	Host	Origin	Genetic Clade [37]	Reference
Brighton Red (AF482758)	Human	UK, Northern Europe	CPXV-like 2	[38]
RatPox09 (LN864565)	Pet rat	Germany, Central Europe	VARV-like	[39]
Ger 91/3 (DQ437593)	Human	Germany, Central Europe	CPXV-like 2	[40]
Ger/2007/Vole (LT896722)	Common vole	Germany, Central Europe	CPXV-like 2	[37]
FM2292 (LN864566)	Common vole	Germany, Central Europe	CPXV-like 2	[33]
Ger/2010/Cat (LT896729)	Cat	Germany, Central Europe	CPXV-like 1	[37]
FIN_MAN_2000 (HQ420893)	Human	Finland, Northern Europe	VACV-like	[41]

CPXV, cowpox virus; VACV, vaccinia virus, VARV, variola virus.

**Table 2:** Design of the animal experiments.

Experiment	Voles			Cowpox Virus				
No./Objective	Lineage	Age	Number of Animals per Group	Strain	Application Route	Dose of Inoculum/Animal	Duration of the Experiment	Link with Results
# 1 susceptibility to different strains	Western lineage	Up to 1 year	9 or 11	Brighton Red Ger/2010/Cat FM2292 Ger/2007/Vole Ger 91/3 RatPox09 FIN_MAN_2000	Intranasal	10^5^ TCID_50_	21 dpi (4/9 or 5/11 animals euthanized for autopsy on 5 dpi)	Table 3 and Table 4 and Table S1
# 2 comparison of different application routes	Western lineage	≈1 year	6 and 2 contacts	RatPox09	Intranasal Subcutaneous	10^5.5^ TCID_50_	14 dpi	Table 5 and Table S2
# 3 comparison of different vole lineages	Western lineage	4 weeks	6 and 3 contacts	RatPox09	Intranasal	10^6^ TCID_50_	14 dpi	Table S3
	Carpathian lineage	4 weeks	6 and 3 contacts	RatPox09	Intranasal	10^6^ TCID_50_	14 dpi	
# 4 comparison of different application routes	Western Lineage	<3 months	6 and 3 contacts	FM2292 RatPox09	Footpad method	10^6^ TCID_50_	28 dpi	Table 6 and Table S4
	Carpathian lineage	<3 months	6 and 3 contacts	FM2292 RatPox09				
# 5 reaction on multiple antigen contact	Mixed	<3 months	6	Brighton Red FM2292 RatPox09	Intranasal (Booster on 21 dpi)	10^5.5^ TCID_50_	42 dpi	Table 7 and Table S5

dpi, days post inoculation; TCID_50_, Tissue Culture InfectiousDose 50.

**Table 3:**
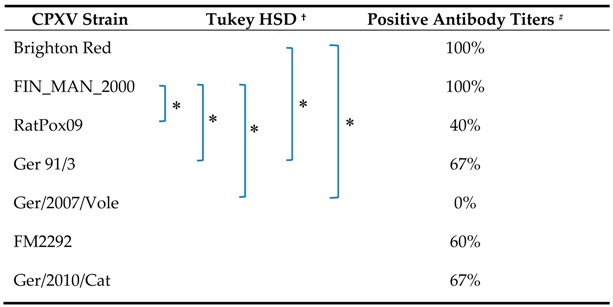
Seroconversion rate of CPXV-inoculated bank voles at 21 dpi.

^†^ Post-hoc-test between the serological reactivity at different dilutions (Table S1) of the different groups for α < 0.05; ^#^ Antibody titers of ≥1:40 were considered positive.

**Table 4:** Viral DNA detection in different organs at 5 dpi.

CPXV Strain	No. Positive/Total No. of Tested Voles *					
	Rhinarium	Trachea	Liver	Spleen	Lung	Skin
Brighton Red	4/5 (1)	4/5 (2)	0/5	0/5	4/10 (4)	1/5 (1)
FIN_MAN_2000	5/5 (1)	3/5 (1)	0/5	0/5	4/10 (0)	0/5
RatPox09	0/4	0/4	0/4	0/4	08	0/4
Ger 91/3	1/4 (1)	2/4 (1)	0/4	0/4	0/8	1/4 (0)
Ger/2007/Vole	1/4 (0)	2/4 (0)	0/4	0/4	0/8	0/4
FM2292	2/4 (0)	2/4 (1)	0/4	0/4	0/8	0/4
Ger/2010/Cat	2/4 (0)	2/4 (1)	0/4	0/4	0/8	0/4

* Cq values of less than 36 were considered positive. Two lung localisations per animal were analysed. Numbers in brackets refer to Cq values below 30, which is considered as positive for replicating virus.

**Table 5:**
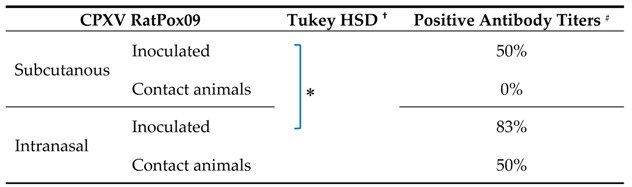
Seroconversion rate of CPXV-inoculated bank voles inoculated with CPXV RatPox09 via different routes, and in contact animals at 14 dpi.

^†^ Post-hoc-test between serological reactivity at different dilutions (Table S2) groups for *p* < 0.05; ^#^ Antibody titers of ≥40 were considered positive.

**Table 6:** Seroconversion rate of bank voles inoculated via the footpad method with either CPXV RatPox09 or CPXV FM2292 (28 dpi); details see Table S4.

Footpad Inoculation		Positive Antibody Titers ^#^
CPXV RatPox09	Inoculated	91.7%
	Contact animals	0%
CPXV FM2292	Inoculated	91.7%
	Contact animals	16.7%

^#^ Antibody titers of ≥1:40 were considered positive.

**Table 7:**
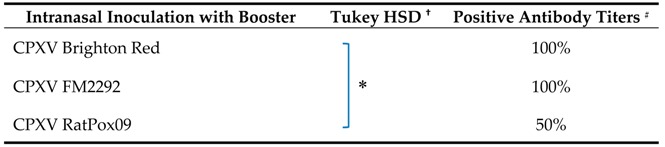
Seroconversion rate of bank voles repeatedly inoculated with the same CPXV strain at 42 dpi.

^†^ Post-hoc-test between serological reactivity at different dilutions (Table S5) groups for *p* <0.05; # Antibody titers of ≥40 were considered positive.

## Supplementary Materials

Supplementary tables are available online at www.mdpi.com/1999-4915/9/12/391/s1.

## References

[1] Kallio-Kokko H., Uzcategui N., Vapalahti O., Vaheri A. (2005). Viral zoonoses in Europe. FEMS Microbiol. Rev..

[2] Wang L.F., Crameri G. (2014). Emerging zoonotic viral diseases. Rev. Sci. Tech..

[3] Popova A.Y., Maksyutov R.A., Taranov O.S., Tregubchak T.V., Zaikovskaya A.V., Sergeev A.A., Vlashchenko I.V., Bodnev S.A., Ternovoi V.A., Alexandrova N.S. (2017). Cowpox in a human, Russia, 2015. Epidemiol. Infect..

[4] Kinnunen P.M., Holopainen J.M., Hemmila H., Piiparinen H., Sironen T., Kivela T., Virtanen J., Niemimaa J., Nikkari S., Jarvinen A. (2015). Severe ocular cowpox in a human, Finland. Emerg. Infect. Dis..

[5] Fassbender P., Zange S., Ibrahim S., Zoeller G., Herbstreit F., Meyer H. (2016). Generalized cowpox virus infection in a patient with HIV, Germany, 2012. Emerg. Infect. Dis..

[6] Switaj K., Kajfasz P., Kurth A., Nitsche A. (2015). Cowpox after a cat scratch—Case report from Poland. Ann. Agric. Environ. Med..

[7] Becker C., Kurth A., Hessler F., Kramp H., Gokel M., Hoffmann R., Kuczka A., Nitsche A. (2009). Cowpox virus infection in pet rat owners: Not always immediately recognized. Dtsch. Arztebl. Int..

[8] Vogel S., Sardy M., Glos K., Korting H.C., Ruzicka T., Wollenberg A. (2012). The Munich outbreak of cutaneous cowpox infection: Transmission by infected pet rats. Acta Derm.-Venereol..

[9] Elsendoorn A., Agius G., Le Moal G., Aajaji F., Favier A.L., Wierzbicka-Hainault E., Beraud G., Flusin O., Crance J.M., Roblot F. (2011). Severe ear chondritis due to cowpox virus transmitted by a pet rat. J. Infect..

[10] Essbauer S., Pfeffer M., Meyer H. (2010). Zoonotic poxviruses. Vet. Microbiol..

[11] Vorou R.M., Papavassiliou V.G., Pierroutsakos I.N. (2008). Cowpox virus infection: An emerging health threat. Curr. Opin. Infect. Dis..

[12] McInerney J., Papasouliotis K., Simpson K., English K., Cook S., Milne E., Gunn-Moore D.A. (2016). Pulmonary cowpox in cats: Five cases. J. Feline Med. Surg..

[13] Goerigk D., Theuß T., Pfeffer M., Konrath A., Kalthoff D., Woll D., Vahlenkamp T.W., Beer M., Starke A. (2014). Kuhpockenvirusinfektion bei einem Alpaka (*Vicugna pacos*)—Klinische Symptomatik, Diagnostik und pathologische Befunde. Tierärztliche Prax. Großtiere.

[14] Kurth A., Wibbelt G., Gerber H.P., Petschaelis A., Pauli G., Nitsche A. (2008). Rat-to-elephant-to-human transmission of cowpox virus. Emerg. Infect. Dis..

[15] Kalthoff D., Bock W.I., Huhn F., Beer M., Hoffmann B. (2014). Fatal cowpox virus infection in cotton-top tamarins (*Saguinus oedipus*) in Germany. Vector-Borne Zoonotic Dis..

[16] Crouch A.C., Baxby D., McCracken C.M., Gaskell R.M., Bennett M. (1995). Serological evidence for the reservoir hosts of cowpox virus in British wildlife. Epidemiol. Infect..

[17] Chantrey J., Meyer H., Baxby D., Begon M., Bown K.J., Hazel S.M., Jones T., Montgomery W.I., Bennett M. (1999). Cowpox: Reservoir hosts and geographic range. Epidemiol. Infect..

[18] Hazel S.M., Bennett M., Chantrey J., Bown K., Cavanagh R., Jones T.R., Baxby D., Begon M. (2000). A longitudinal study of an endemic disease in its wildlife reservoir: Cowpox and wild rodents. Epidemiol. Infect..

[19] Boulanger D., Crouch A., Brochier B., Bennett M., Clement J., Gaskell R.M., Baxby D., Pastoret P.P. (1996). Serological survey for orthopoxvirus infection of wild mammals in areas where a recombinant rabies virus is used to vaccinate foxes. Vet. Rec..

[20] Pelkonen P.M., Tarvainen K., Hynninen A., Kallio E.R., Henttonen K., Palva A., Vaheri A., Vapalahti O. (2003). Cowpox with severe generalized eruption, Finland. Emerg. Infect. Dis..

[21] Kinnunen P.M., Henttonen H., Hoffmann B., Kallio E.R., Korthase C., Laakkonen J., Niemimaa J., Palva A., Schlegel M., Ali H.S. (2011). Orthopox virus infections in Eurasian wild rodents. Vector-Borne Zoonotic Dis..

[22] Tryland M., Sandvik T., Mehl R., Bennett M., Traavik T., Olsvik O. (1998). Serosurvey for orthopoxviruses in rodents and shrews from Norway. J. Wildl. Dis..

[23] Essbauer S., Hartnack S., Misztela K., Kiessling-Tsalos J., Baumler W., Pfeffer M. (2009). Patterns of orthopox virus wild rodent hosts in South Germany. Vector-Borne Zoonotic Dis..

[24] Marennikova S.S., Ladnyj I.D., Ogorodinikova Z.I., Shelukhina E.M., Maltseva N.N. (1978). Identification and study of a poxvirus isolated from wild rodents in Turkmenia. Arch. Virol..

[25] Van Cuong N., Carrique-Mas J., Vo Be H., An N.N., Tue N.T., Anh N.L., Anh P.H., Phuc N.T., Baker S., Voutilainen L. (2015). Rodents and risk in the Mekong delta of Vietnam: Seroprevalence of selected zoonotic viruses in rodents and humans. Vector-Borne Zoonotic Dis..

[26] Tsanava S.A., Sakvarelidze L.A., Shelukhina E.M. (1989). Serologic survey of wild rodents in Georgia for antibodies to orthopoxviruses. Acta Virol..

[27] Oldal M., Sironen T., Henttonen H., Vapalahti O., Madai M., Horvath G., Dallos B., Kutas A., Foldes F., Kemenesi G. (2015). Serologic survey of orthopoxvirus infection among rodents in Hungary. Vector-Borne Zoonotic Dis..

[28] Begon M., Hazel S.M., Baxby D., Bown K., Cavanagh R., Chantrey J., Jones T., Bennett M. (1999). Transmission dynamics of a zoonotic pathogen within and between wildlife host species. Proc. R. Soc. Lond. B Biol. Sci..

[29] Turner A.K., Beldomenico P.M., Bown K., Burthe S.J., Jackson J.A., Lambin X., Begon M. (2014). Host-parasite biology in the real world: The field voles of Kielder. Parasitology.

[30] Beldomenico P.M., Telfer S., Lukomski L., Gebert S., Bennett M., Begon M. (2009). Host condition and individual risk of cowpox virus infection in natural animal populations: Cause or effect?. Epidemiol. Infect..

[31] Bennett M., Crouch A.J., Begon M., Duffy B., Feore S., Gaskell R.M., Kelly D.F., McCracken C.M., Vicary L., Baxby D. (1997). Cowpox in British voles and mice. J. Comp. Pathol..

[32] Feore S.M., Bennett M., Chantrey J., Jones T., Baxby D., Begon M. (1997). The effect of cowpox virus infection on fecundity in bank voles and wood mice. Proc. R. Soc. Lond. B Biol. Sci..

[33] Hoffmann D., Franke A., Jenckel M., Tamosiunaite A., Schluckebier J., Granzow H., Hoffmann B., Fischer S., Ulrich R.G., Hoper D. (2015). Out of the reservoir: Phenotypic and genotypic characterization of a novel cowpox virus isolated from a common vole. J. Virol..

[34] Aguirre A.A., Ostfeld R.S., Daszak P. (2012). New Directions in Conservation Medicine: Applied Cases of Ecological Health.

[35] Filipi K., Markova S., Searle J.B., Kotlik P. (2015). Mitogenomic phylogenetics of the bank vole *Clethrionomys glareolus*, a model system for studying end-glacial colonization of Europe. Mol. Phylogenet. Evol..

[36] Drewes S., Ali H.S., Saxenhofer M., Rosenfeld U.M., Binder F., Cuypers F., Schlegel M., Rohrs S., Heckel G., Ulrich R.G. (2017). Host-associated absence of human *Puumala virus* infections in Northern and Eastern Germany. Emerg. Infect. Dis..

[37] Franke A., Pfaff F., Jenckel M., Hoffmann B., Hoper D., Antwerpen M., Meyer H., Beer M., Hoffmann D. (2017). Classification of cowpox viruses into several distinct clades and identification of a novel lineage. Viruses.

[38] Downie A.W. (1939). A study of the lesions produced experimentally by cowpox virus. J. Pathol. Bacteriol..

[39] Kalthoff D., Konig P., Meyer H., Beer M., Hoffmann B. (2011). Experimental cowpox virus infection in rats. Vet. Microbiol..

[40] Meyer H., Schay C., Mahnel H., Pfeffer M. (1999). Characterization of orthopoxviruses isolated from man and animals in Germany. Arch. Virol..

[41] Carroll D.S., Emerson G.L., Li Y., Sammons S., Olson V., Frace M., Nakazawa Y., Czerny C.P., Tryland M., Kolodziejek J. (2011). Chasing Jenner’s vaccine: Revisiting cowpox virus classification. PLoS ONE.

[42] Schlegel M., Ali H.S., Stieger N., Groschup M.H., Wolf R., Ulrich R.G. (2012). Molecular identification of small mammal species using novel *cytochrome b* gene-derived degenerated primers. Biochem. Genet..

[43] Maksyutov R.A., Gavrilova E.V., Meyer H., Shchelkunov S.N. (2015). Real-time PCR assay for specific detection of cowpox virus. J. Virol. Methods.

[44] Wolfs T.F., Wagenaar J.A., Niesters H.G., Osterhaus A.D. (2002). Rat-to-human transmission of cowpox infection. Emerg. Infect. Dis..

[45] Shchelkunov S.N., Marennikova S.S., Moyer R.W. (2005). Orthopoxviruses Pathogenic for Humans.

[46] Miller R.E., Fowler M. (2011). Fowler's Zoo and Wild Animal Medicine Current Therapy.

[47] Shenbrot G.I., Krasnov B.R. (2005). An Atlas of the Geographic Distribution of the Arvicoline Rodents of the World (Rodentia, Muridae: Arvicolinae).

[48] Haydon D.T., Cleaveland S., Taylor L.H., Laurenson M.K. (2002). Identifying reservoirs of infection: A conceptual and practical challenge. Emerg. Infect. Dis..

[49] Plourde B.T., Burgess T.L., Eskew E.A., Roth T.M., Stephenson N., Foley J.E. (2017). Are disease reservoirs special? Taxonomic and life history characteristics. PLoS ONE.

[50] Humair P.F., Rais O., Gern L. (1999). Transmission of *Borrelia afzelii* from *Apodemus* mice and *Clethrionomys* voles to *Ixodes ricinus* ticks: Differential transmission pattern and overwintering maintenance. Parasitology.

