# Supplementary material for: Experimental Cowpox Virus (CPXV) Infections of Bank Voles: Exceptional Clinical Resistance and Variable Reservoir Competence
Source: Viruses. 2017 Dec 19;9(12):391. doi: 10.3390/v9120391 (PMC5744165; doi:10.3390/v9120391)
Supplement: Supplementary file 1 [file viruses-09-00391-s001.pdf]

# Supplemental material:

## Table S1

Detection and titration of OPV-specific antibodies in vole sera taken at 21 dpi (intranasal inoculation)

| | **Antibody titers (No. of positive/total no. of tested voles)*** | | | | |
|---|---|---|---|---|---|
| **CPXV strain** | **20** | **40** | **80** | **160** | **320** |
| Brighton Red | **4/4** | **4/4** | 4/4 | 3/4 | 0/4 |
| FIN_MAN_2000 | **6/6** | **6/6** | 5/6 | 5/6 | 1/6 |
| RatPox09 | 4/5 | 2/5 | 1/5 | 1/5 | 0/5 |
| Ger 91/3 | 4/6 | 4/6 | 0/6 | 0/6 | 0/6 |
| Ger/2007/Vole | 1/5 | 0/5 | 0/5 | 0/5 | 0/5 |
| FM2292 | 4/5 | 3/5 | 3/5 | 1/5 | 0/5 |
| Ger/2010/Cat | 2/3 | 2/3 | 2/3 | 1/3 | 0/3 |

*Antibody titers of ≥ 40 were considered positive.

**Table S2**

Antibody titers in bank voles inoculated with CPXV via different routes and in contact animals at 14 dpi

| CPXV RatPox09 | | Antibody titers (no. of positive/total no. of animals tested)* | | | | |
|---|---|---|---|---|---|---|
| | | **20** | **40** | **80** | **160** | **320** |
| **Subcutanous** | **Inoculated** | **4/6** | **3/6** | **2/6** | **1/6** | **1/6** |
| | **Contact animals** | 0/1 | 0/1 | 0/1 | 0/1 | 0/1 |
| **Intranasal** | **Inoculated** | **5/6** | **5/6** | **5/6** | **5/6** | **5/6** |
| | **Contact animals** | **1/2** | **1/2** | **1/2** | **1/2** | **1/2** |

*Antibody titers of ≥ 40 were considered positive.

## Table S3

Antibody titers in 4-weeks old bank voles from Western and Carpathian lineage inoculated with CPXV RatPox09 at 14 dpi

| **Intranasal inoculation with CPXV RatPox09** | | **Antibody titers (no. of positive/total no. of animals tested)*** | |
|---|---|---|---|
| | | **20** | **80** |
| **Western lineage** | **Inoculated** | **5/5** | **1/5** |
| | **Contact animals** | 0/3 | 0/3 |
| **Carpathian lineage** | **Inoculated** | **6/6** | **1/6** |
| | **Contact animals** | 0/3 | 0/3 |

*Antibody titers of ≥ 40 were considered positive.

## Table S4

Antibody titers in bank voles inoculated via the footpad method with either CPXV RatPox09 or CPXV FM2292 (28 dpi)

| **Footpad inoculation** | | **Antibody titers (no. of positive/total no. of voles tested)*** | | | | |
|---|---|---|---|---|---|---|
| | | **20** | **40** | **80** | **160** | **320** |
| **CPXV RatPox09** | **Inoculated** | **11/12** | **8/12** | **6/12** | **4/12** | **1/12** |
| | **Contact animals** | 0/4 | 0/4 | 0/4 | 0/4 | 0/4 |
| **CPXV FM2292** | **Inoculated** | **11/12** | **11/12** | **10/12** | **7/12** | **1/12** |
| | **Contact animals** | **1/6** | **1/6** | **1/6** | **1/6** | **1/6** |

*Antibody titers of ≥ 40 were considered positive.

## Table S5

Antibody titers of bank voles repeatedly inoculated with the same CPXV strain at 42 dpi

| Intranasal inoculation with booster | Antibody titers (no. of positive/total no. of voles tested)* | | | | |
|---|---|---|---|---|---|
| | 20 | 40 | 80 | 160 | 320 |
| **CPXV Brighton Red** | **6/6** | **6/6** | **6/6** | **6/6** | **6/6** |
| **CPXV FM2292** | **6/6** | **6/6** | **6/6** | **6/6** | **1/6** |
| **CPXV RatPox09** | 3/6 | 3/6 | 3/6 | 3/6 | 1/6 |

*Antibody titers of ≥ 40 were considered positive.
